# Pan-cancer investigation of C-to-U editing reveals its important role in cancer development and new targets for cancer treatment

**DOI:** 10.3389/fonc.2023.1097667

**Published:** 2023-03-09

**Authors:** Mengbiao Guo, Feng Li, Linghao Zhao, Zhengwen Fang, Huichuan Yu, Zhou Songyang, Yuanyan Xiong

**Affiliations:** ^1^ Key Laboratory of Gene Engineering of the Ministry of Education, Institute of Healthy Aging Research, School of Life Sciences, Sun Yat-sen University, Guangzhou, China; ^2^ Eastern Hepatobiliary Surgery Hospital, Second Military Medical University, Shanghai, China; ^3^ Department of Colorectal Surgery, The Sixth Affiliated Hospital, Sun Yat-sen University, Guangzhou, Guangdong, China

**Keywords:** pan-cancer C-to-U editing, tumor-infiltrated immune cells, cancer stemness, immune checkpoint blockade, colon cancer, *CST3*, *CSNK2B*, *RPS14*

## Abstract

RNA editing is prevalent in the transcriptome and is important for multiple cellular processes. C-to-U RNA editing sites (RES) are relatively rare and understudied in humans, compared to A-to-I editing. However, the functional impact of C-to-U editing in human cancers also remains elusive. Here, we conducted the first comprehensive survey of pan-cancer C-to-U RESs. Surprisingly, we found that the same subset of RESs were associated with multiple features, including patient survival, cancer stemness, tumor mutation burden (TMB), and tumor-infiltrated immune cell compositions (ICC), suggesting an RES-mediated close relationship between these features. For example, editing sites for GALM or IFI6 that led to higher expression were linked to lower survival and more cancer stemness. Also, TMB was found to be lower in prostate cancer cases with ICC-associated RESs in CAVIN1 or VWA8 or higher in prostate cancer cases with thymoma. With experimental support, we also found RESs in CST3, TPI1, or TNC that are linked to immune checkpoint blockade by anti-PD1. We also confirmed through experiments that two C-to-U RESs in CSNK2B or RPS14 had different effects on colon cancer cells. Patients with CSNK2B editing, which increased the expression of the oncogene CLDN18, had a lower response to drugs. On the other hand, drugs worked better on people who had RPS14 editing, which greatly increased ribosome production. In summary, our study demonstrated the important roles of C-to-U RESs across cancers and shed light on personalized cancer therapy.

## Introduction

Millions of RNA editing sites exist in the human transcriptome (1, 2), including mainly adenosine-to-inosine (A-to-I) by ADARs and less frequent cytosine-to-uridine (C-to-U) by APOBECs (3). As a type of pervasive epigenetic or epitranscriptomic modification, it does not affect DNA sequence identity, but could contribute to protein diversity if located in coding regions (4). Interestingly, several functional A-to-I events have been reported in cancer, such as AZIN1 editing in liver and colorectal cancer (5, 6) and RHOQ editing in colorectal cancer (7). Since the launch of The Cancer Genome Atlas (TCGA), several studies have identified clinically relevant A-to-I editing sites across cancer types by using RNA sequencing (RNA-seq) data (4, 8, 9).

However, the research on C-to-U editing has been largely lagging behind, especially in cancer (3), although scattered reports have studied the function of APOBEC enzymes, which are responsible for most C-to-U editing, and their cofactors (10–12). The first and most famous C-to-U editing was in apolipoprotein B mRNA in the small intestine, which gave APOBEC its name (13). Compared to A-to-I editing, only a very limited number of C-to-U RESs have been identified so far, mostly for highly edited genes or under unnatural conditions, and without functional validation in disease following identification (14–18). Moreover, the functions of most C-to-U editing sites remain unclear. To our knowledge, no C-to-U editing has been demonstrated to contribute to tumor pathogenesis in most human cancers (3).

Here, we conducted the first systematic survey of C-to-U RESs across >20 cancer types. A subset of these RESs were associated with patient survival, cancer stemness, tumor mutation burden (TMB), tumor-infiltrated immune cell compositions (ICCs) in the tumor microenvironment, and even responses to anti-PD1 immunotherapy treatment. Importantly, we experimentally validated two C-to-U RESs, in *CSNK2B* (casein kinase II beta subunit) and *RPS14* (ribosomal protein S14), respectively, with different impacts on cell proliferation in colon cancer.

## Results

### Overview of C-to-U RNA editing across cancer types

**Figure 1:**
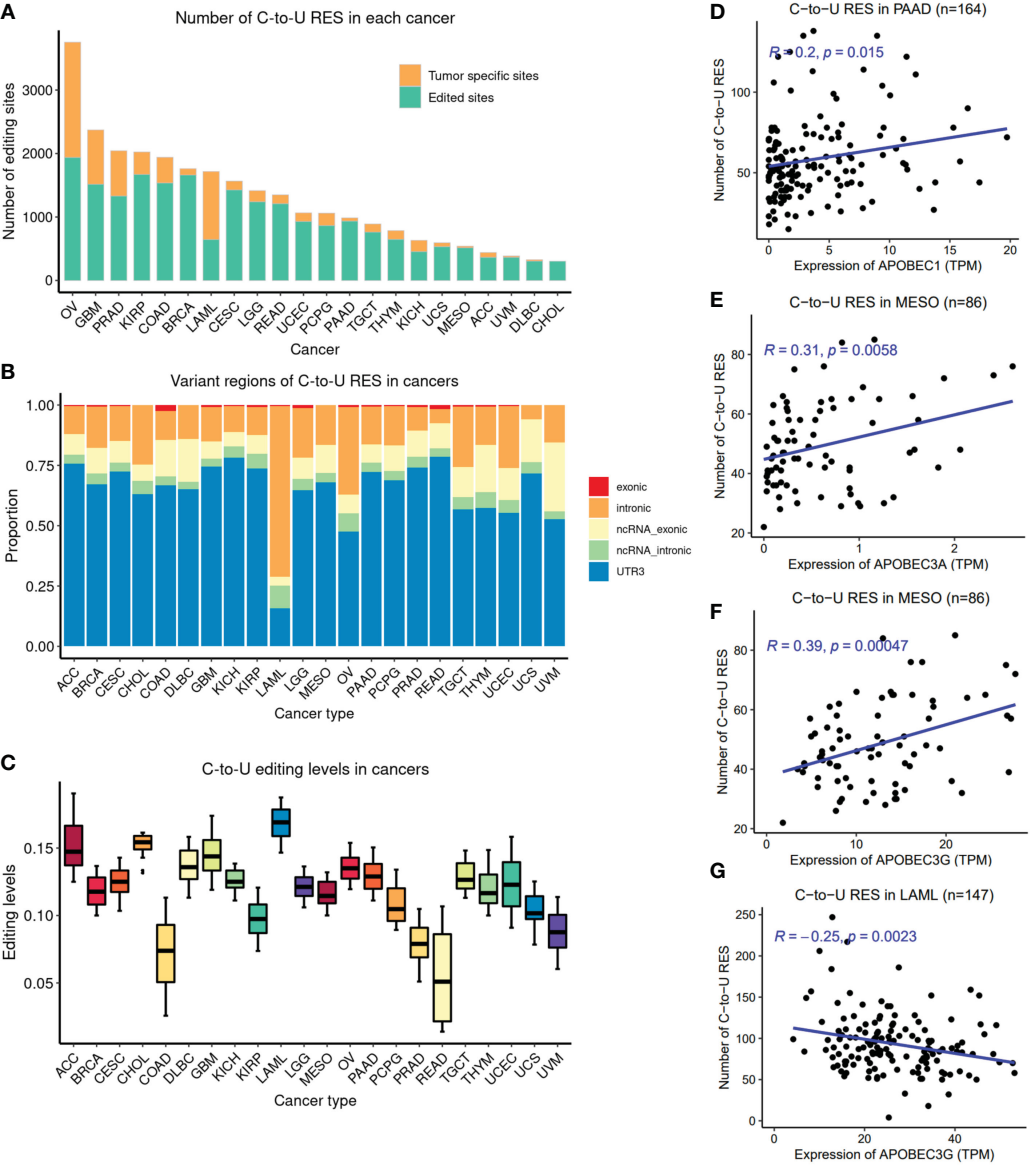
Overview of pan-cancer C-to-U RESs. **(A)** Number of RESs (nRES) across cancer types. The orange color indicates tumor-type-specificity. **(B)** Genomic region distribution of RESs across cancers. LAML was derived from blood, in contrast to other solid tumors. **(C)** Overall editing levels of RESs across cancers. The overall editing level for each sample is defined as the median editing level of RESs. **(D)** Positive correlation between nRES and *APOBEC1* expression in PAAD, as an example. **(E)** Positive correlation between nRES and *APOBEC3A* expression in MESO, as an example. **(F)** Positive correlation between nRES and *APOBEC3G* expression in MESO, as an example. **(G)** Negative correlation between nRES and *APOBEC3G* expression in LAML. ACC, Adrenocortical carcinoma; BRCA, Breast invasive carcinoma; CESC, Cervical squamous cell carcinoma and endocervical adenocarcinoma; CHOL, Cholangiocarcinoma; COAD, Colon Adenocarcinoma; DLBC, Lymphoid Neoplasm Diffuse Large B-cell Lymphoma; GBM, Glioblastoma multiforme; KICH, Kidney Chromophobe; KIRP, Kidney renal papillary cell carcinoma; LAML, Acute Myeloid Leukemia; LGG, Brain Lower Grade Glioma; MESO, Mesothelioma; OV, Ovarian serous cystadenocarcinoma; PAAD, Pancreatic adenocarcinoma; PCPG, Pheochromocytoma and Paraganglioma; PRAD, Prostate adenocarcinoma; READ, Rectum adenocarcinoma; TGCT, Testicular Germ Cell Tumors; THYM, Thymoma; UCEC, Uterine Corpus Endometrial Carcinoma; UCS, Uterine Carcinosarcoma; UVM, Uveal Melanoma.

First, we obtained pan-cancer (N=22 cancer types) C-to-U editing sites (see **Methods**). After quality control (see **Methods**), we obtained 45,4185 editing events in samples from 22 cancer types, which resulted in 10,595 unique editing sites in the transcriptome (**Figure 1A**). Ovarian cancer (OV) had the highest number of RESs (nRES >3,500; referring to C-to-U unless otherwise specified), followed by glioma (GBM), prostate cancer (PRAD), kidney cancer (KIRP), colon cancer (COAD), breast cancer (BRCA), and leukemia (LAML), which should be partly due to the number of available samples for each cancer. The distribution of C-to-U events was different from A-to-I events (**Figure S1A**), for which BRCA was comparable to OV.

Interestingly, most RESs in tumor samples were shared by at least two cancer types, but half the RESs of OV and ~60% of the RESs of LAML were tumor-type-specific. The majority of RESs were found in the 3’UTR, followed by introns (**Figure 1B**); however, LAML RESs were mostly found in introns (mRNAs or noncoding RNAs), probably because it is the only blood cancer here, as opposed to other solid tumors. This pattern was also observed for A-to-I events, obtained from the previous study of A-to-I editing sites by our group (19) (**Figure S1B**). Moreover, LAML RESs showed the highest editing levels (EL) among all cancer types, while COAD and READ showed the lowest ELs (**Figure 1C**). However, this pattern was not observed in A-to-I events (**Figure S1C**). Of note, within brain cancers, GBM (advanced or malignant glioma) had higher ELs than LGG (lower-grade glioma), while for kidney cancers, KICH had higher ELs than KIRP. Surprisingly, we observed significant negative associations between EL and the number of RESs (nRES) in 14 out of 22 cancer types (**Figure S2**), suggesting a potential balance between nRES and EL in these cancers.

Three APOBEC family members, APOBEC1, APOBEC3A, and APOBEC3G, are likely to have contributed the greatest number of C-to-U RESs (3, 14). We examined the correlation between their expression levels and nRES. Of note, *APOBEC1* and *APOBEC3A* expression were only detected in a handful of cancers (**Figures S1D-E**), such as *APOBEC1* in PAAD, in contrast to *APOBEC3G*, which was detected in most cancer types (**Figure S1F**). *APOBEC1* was positively correlated with nRES in PAAD (**Figure 1D**). A positive *APOBEC3A* correlation with nRESs was detected mainly in BRCA, LGG, PAAD, and MESO (**Figure 1E**). Interestingly, *APOBEC3G* was positively correlated with nRESs in multiple cancer types, such as CESC, PCPG, THYM, and MESO (**Figure 1F**), but was negatively correlated in LAML (**Figure 1G**), for unknown reasons.

### C-to-U editing predicts poor survival of patients with multiple cancer types

**Figure 2:**
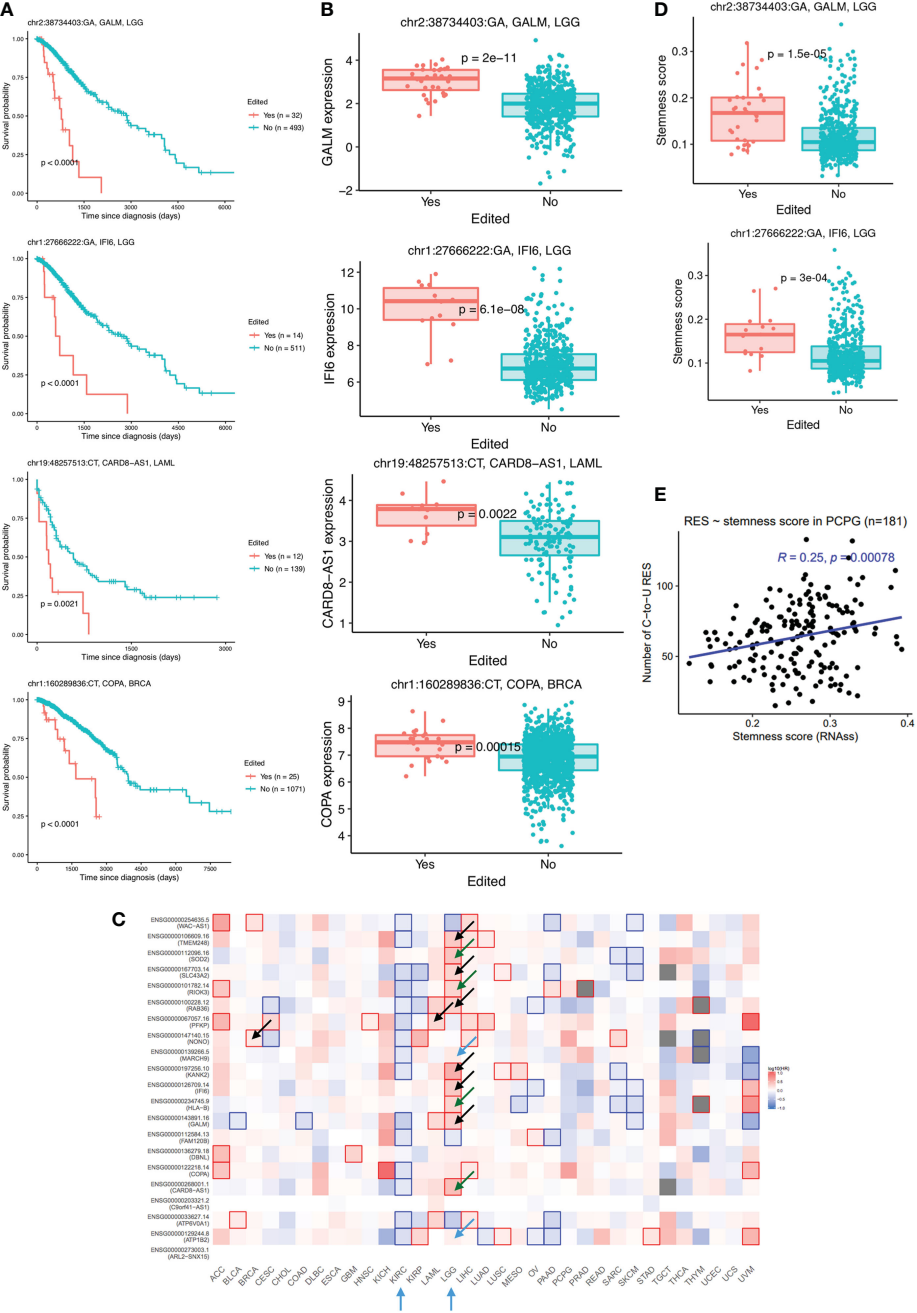
Consistent association between C-to-U RESs and various tumor features. **(A)** Worse survival of patients with C-to-U editing of GALM and IFI6 in LGG, CARD8-AS1 in LAML, and COPA in BRCA. **(B)** RESs shown in **(A)** resulted in higher expression levels of corresponding genes. **(C)** Expression levels of most genes with survival-associated RESs were themselves prognostic for patients, mostly in LGG or KIRC, as indicated by arrows. Arrows were colored as follows: black (*P*<0.05) and blue (suggestive *P*-values) for matched cancers where RESs were identified; green for different cancers from the one where RES were identified. **(D)** Two RESs shown in **(A)**, GALM and IFI6 in LGG, were associated with higher cancer stemness. **(E)** Positive correlation between cancer stemness (RNAss, or RNA stemness score) and nRES in PCPG, as an example.

To explore the clinical significance of C-to-U RESs, we examined their association with patient survival. A total of 28 RESs (from 21 genes), mostly located in introns or 3’UTRs, were found to be prognostic for patient outcomes of multiple cancer types (**Table S1**), including 15 RESs in LGG and five in LAML. Notably, two RESs from LGG were found in the 3’UTR of *GALM* (galactose mutarotase) and another in the 3’UTR of *IFI6* (interferon alpha inducible protein 6). Three RESs from LAML were found in the intron of *FAM120B* (constitutive coactivator of peroxisome proliferator-activated receptor gamma), and two RESs from BRCA were found in the 3’UTR of *COPA* (coatomer subunit alpha) (**Figure 2A**). Of note, A-to-I editing sites in COPA have already been associated with multiple cancer types, including BRCA (4) and liver cancer (HCC) (20). Remarkably, for each of the 28 RESs, patients with the editing event showed much worse survival than other patients with cancer. Moreover, 16 out of those 28 RESs, located in 12 genes, were associated with higher expression of the edited genes (**Figure 2B**), and one RES was associated with lower gene expression (*HLA-B* in BRCA). Consistently, higher expression of 10 out of 12 host genes of the above 16 RESs predicted worse patient survival (eight significant and two suggestive prognosis *P*-values) (**Figure 2C**). Interestingly, higher expression of most of the 21 genes, including *HLA-B*, predicted significantly worse outcomes in LGG but better outcomes in KIRC. These results support important functions of C-to-U RESs across multiple cancer types.

### C-to-U editing is positively correlated with cancer stemness

The potential existence of cancer stem cells (21), as reflected by cancer stemness (22), is probably the main reason for cancer aggressiveness and why most cancers relapse. Interestingly, we found that four of the 28 prognostic RESs were associated with higher stemness in corresponding cancers, including RESs of *GALM* and *IFI6* in LGG (**Figure 2D**). We further examined the global association between RESs and cancer stemness. We found significant positive correlations between nRES and RNA stemness scores (RNAss) in BRCA, PCPG, TGCT, and UCEC cancers (**Figure 2E**).

Cancer stemness is closely associated with the microenvironment (23). A previous study has identified six immune subtypes across TCGA cancer types (24). We found a significant difference in EL among immune subtypes in four cancer types, BRCA, PCPG, TGCT, and UCS (**Figure S3**). Consistently, we found that Immune C1 [Wound Healing, which is closely associated with high cancer stemness (25)] showed higher EL than Immune C2 (IFN-gamma Dominant) (two-sided Wilcoxon test, *P*=0.042 in TGCT, *P*=0.031 in UCS, and *P*<0.001 in BRCA), and Immune C3 [Inflammatory, which can enhance cancer stemness (25)] showed higher EL than Immune C4 (Lymphocyte Depleted) (two-sided Wilcoxon test, *P*<0.01 in both BRCA and in PCPG) (**Figure S3**).

### C-to-U editing bridges tumor immune infiltration and TMB

**Figure 3:**
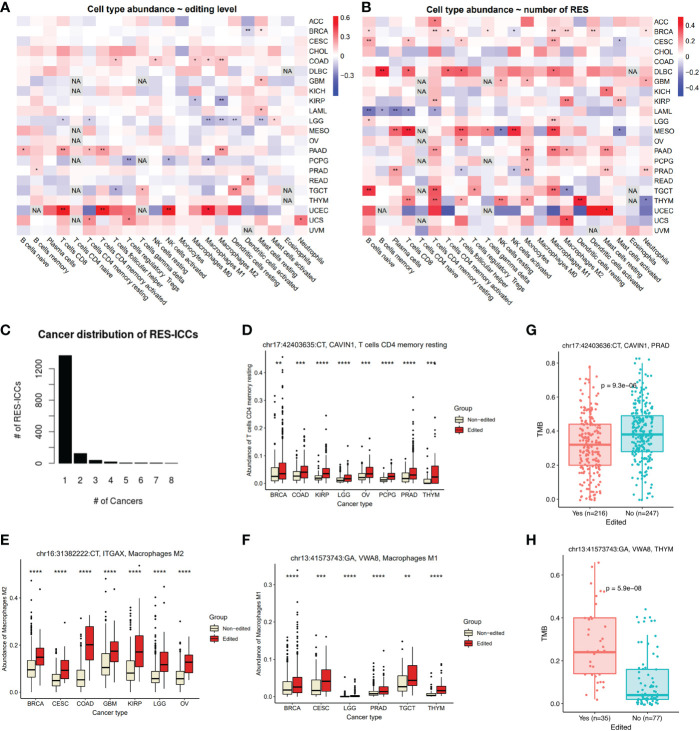
Association between RESs and ICCs or TMB across cancers. **(A, B)** Pearson correlation between ICCs and editing levels **(A)** or numbers of RESs **(B)** in each cancer. Colors were correlation coefficient and asterisks were significant. **(C)** Distribution of number of cancers with the same RES-ICC associations. **(D–F)** An example of RES-ICC of *CAVIN1* associated with CD4+ T cells **(D)**, *ITGAX* with M2 macrophages **(E)**, and *VWA8* with M1 macrophages **(F)** across cancers. Samples were grouped by editing status. **(G)** ICC-associated RESs in *CAVIN1* were also associated with lower TMB levels in PRAD. **(H)** ICC-associated RESs in *VWA8* were also associated with higher TMB levels in THYM. *P<0.05, **P<0.01, ***P<0.001, ****P<0.0001. Mann-Withney-Wilcoxon test was used for two-group comparison. NA, not available.

Based on the above EL associations with cancer immune subtypes, we hypothesized that RES would probably be associated with infiltrated immune cells or immune cell compositions (ICC) in tumors. Indeed, we found that ELs were mostly associated with at least one type of ICCs (**Figure 3A**). Notably, UCEC showed the strongest correlation between ELs and multiple ICCs, including CD8+ T cells, activated CD4+ memory T cells, activated NK cells, and M1 macrophages. PAAD and COAD also showed mostly positive correlations between ELs and multiple ICCs, in contrast to LGG, PCPG, and KIRP. On the other hand, most ICCs showed positive correlations with nRES across cancer types, especially in BRCA, PAAD, DLBC, and THYM, except for LAML (**Figure 3B**).

Next, we investigated associations between ICCs and individual RESs. In total, we identified 1,562 significant RES-ICC associations, with identical RES-ICC associations appearing in as many as eight cancer types (**Figure 3C**, **Table S2**). We found that multiple RESs in *CAVIN1* 3’UTR were correlated with resting CD4+ memory T cells in at least six cancer types (**Figure 3D**), seven in *IGAX* 3’UTR (**Figure 3E**) with M2 macrophages in at least six cancer types, and one in *VWA8* intron with M1 macrophages in six cancer types (**Figure 3F**).

Interestingly, three ICC-associated RESs of *CAVIN1* (Caveolae Associated Protein 1) were also associated with lower TMB in PRAD (**Figure 3G**). Six ICC-associated RESs of *VWA8* (Von Willebrand Factor A Domain Containing 8) were also associated with higher TMB in THYM (**Figure 3H**). We further explored the overall connections between ICC-RESs and TMB-RESs. As a result, we identified a total of 84 RESs from 37 genes, such as *C7, CD24, ICAM1, MUC3A, PPP1R3B, PRR11, PSMA8*, and *TTF2*, associated with both ICCs and TMBs (**Table S2**). RESs may affect gene expression of TMB regulators, which subsequently affects neoantigens presented on the tumor cell surface and influences ICCs.

### C-to-U editing reveals both known and novel mechanisms underlying anti-PD1 immunotherapy response

**Figure 4:**
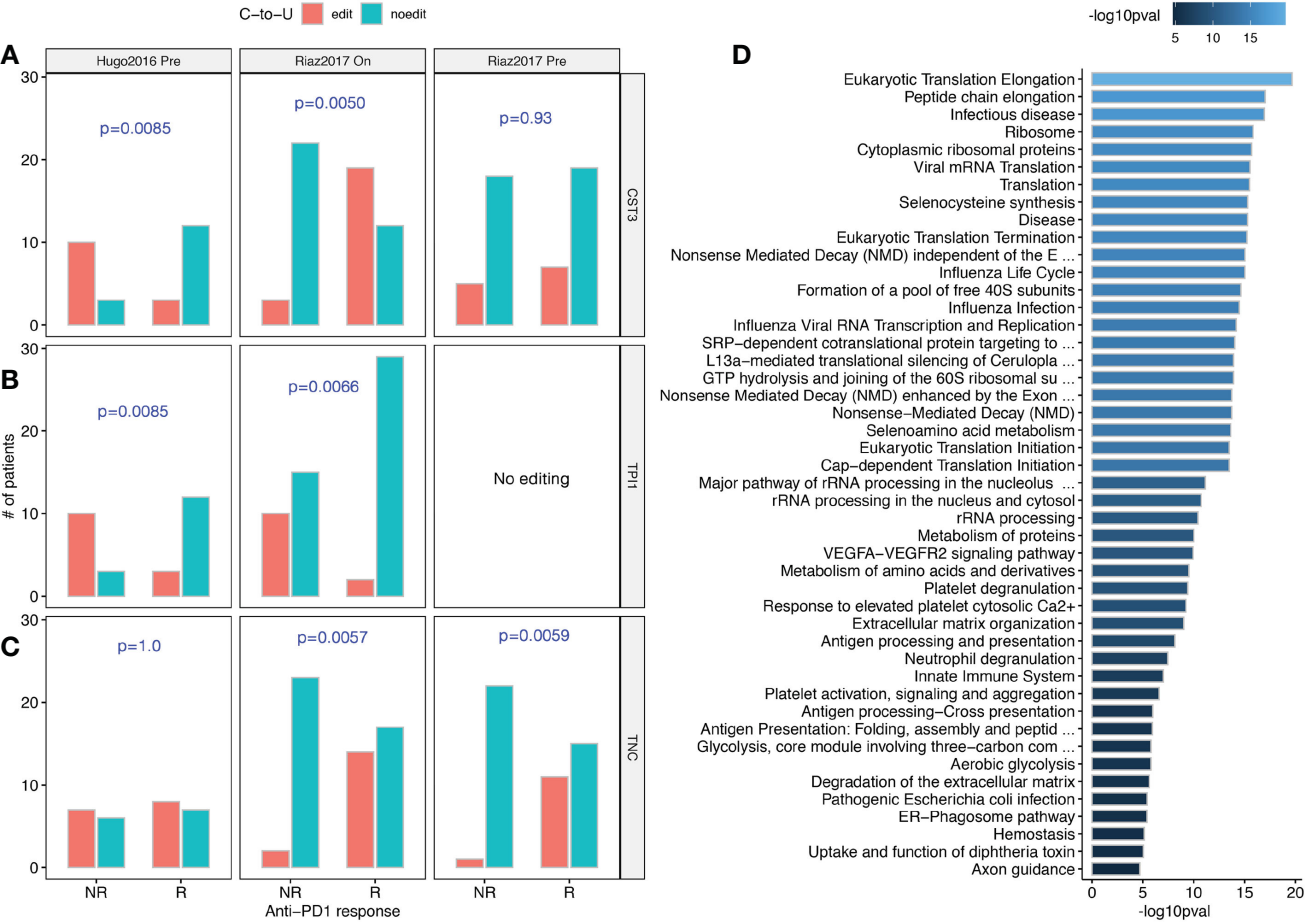
C-to-U RESs associated with responses to anti-PD1 immunotherapy treatment. **(A)** Differential editing of *CST3*, a verified modulator of anti-PD1 response, were observed in both Hugo2016 and Riaz2017 datasets. **(B)** Differential editing of *TPI1*, a suggestive effector of the anti-PD1 response, was observed in both the Hugo2016 and Riaz2017 datasets. **(C)** Differential editing of *TNC*, a reported synergistic cofactor of anti-PD1 response, were observed in both before and during anti-PD1 treatment of the Riaz2017 dataset. **(D)** Pathway enrichment for genes with anti-PD1 response-associated RESs and shared between both Hugo2016 and Riaz2017 datasets. Bar colors were -log10 *P*-values of enrichment. **(A–C)** One-sided chi-squared test.

TMB has been proposed as an important FDA-approved biomarker of immunotherapy based on immune checkpoint inhibitors (ICIs) (26). Naturally, we proceed to investigate the relationship between C-to-U RESs and anti-PD1 immunotherapy. Two melanoma cohorts with anti-PD1 treatment were examined (see **Methods**). Interestingly, we observed that two anti-PD1 response-associated RESs (*P*<0.01), both exonic, were shared between the two cohorts. The first RES, chr20:23635344 (**Figure 4A**), was in the *CST3* gene that encodes Cystatin C, an inhibitor of cysteine proteases. *CST3* editing was more frequent in patients responsive to anti-PD1 during treatment. Notably, *CST3* was recently reported as a glucocorticoid response gene and can predict immunotherapy failure of patients with multiple cancers, including melanoma, possibly by directing recruitment of Trem2+ macrophages (27). In cancer, CST3 has also been linked to the removal of T cells and a lack of response to blocking immune checkpoints (28). Therefore, our results suggest that *CST3* RES probably affected ICI treatment response in melanoma patients.

The other RES, chr12:6870372 (**Figure 4B**), was in the *TPI1* gene that encodes triosephosphate isomerase 1, a very efficient metabolic enzyme in glycolysis and gluconeogenesis. Cancer cells preferentially use glycolysis instead of mitochondrial oxidative phosphorylation to produce energy, which is known as the Warburg Effect (29). Lactic acid, the glycolytic by-product, can facilitate suppression of infiltrating effector T cells by metabolically reprogramming regulatory T cells in tumors (30). *TPI1* editing was almost absent in patients responsive to anti-PD1 treatment. Although not reported as an immunotherapy target, *TPI1* was found among the transcriptional programs driven by anti-PD1 treatment (31). Therefore, *TPI1* RES probably also affected ICI treatment response in melanoma patients.

Furthermore, we noticed that a RES in *TNC* (tenascin C, chr9:115021114) was associated with patient responses both before and during anti-PD1 treatment (**Figure 4C**). TNC induces autophagy-mediated immunosuppression and its blockade can boost ICI treatment efficacy (32). Lastly, when studying RESs aggregated at the gene level, we observed 116 genes were significant in both anti-PD1 patient cohorts. **Figure 4D** shows that these genes were more common in areas like infectious diseases, ribosomes or translation, antigen processing and presentation, and glycolysis.

### C-to-U editing sites in CSNK2B and RPS14 have distinct effects on colon cancer cells

Colorectal cancer is among the most common and deadliest cancer types and presents considerable molecular heterogeneity (33). Next, we screened colon cancer RESs for experimental validation, based on a series of filters (see **Methods**). We only considered missense or nonsense RESs in coding regions. We also took out RESs from the RADAR database (1) to avoid those that had already been profiled and looked at in cancer (4, 8, 9). In the end, we characterized two of the final thirteen RESs and experimentally validated their impact on colon cancer.

**Figure 5:**
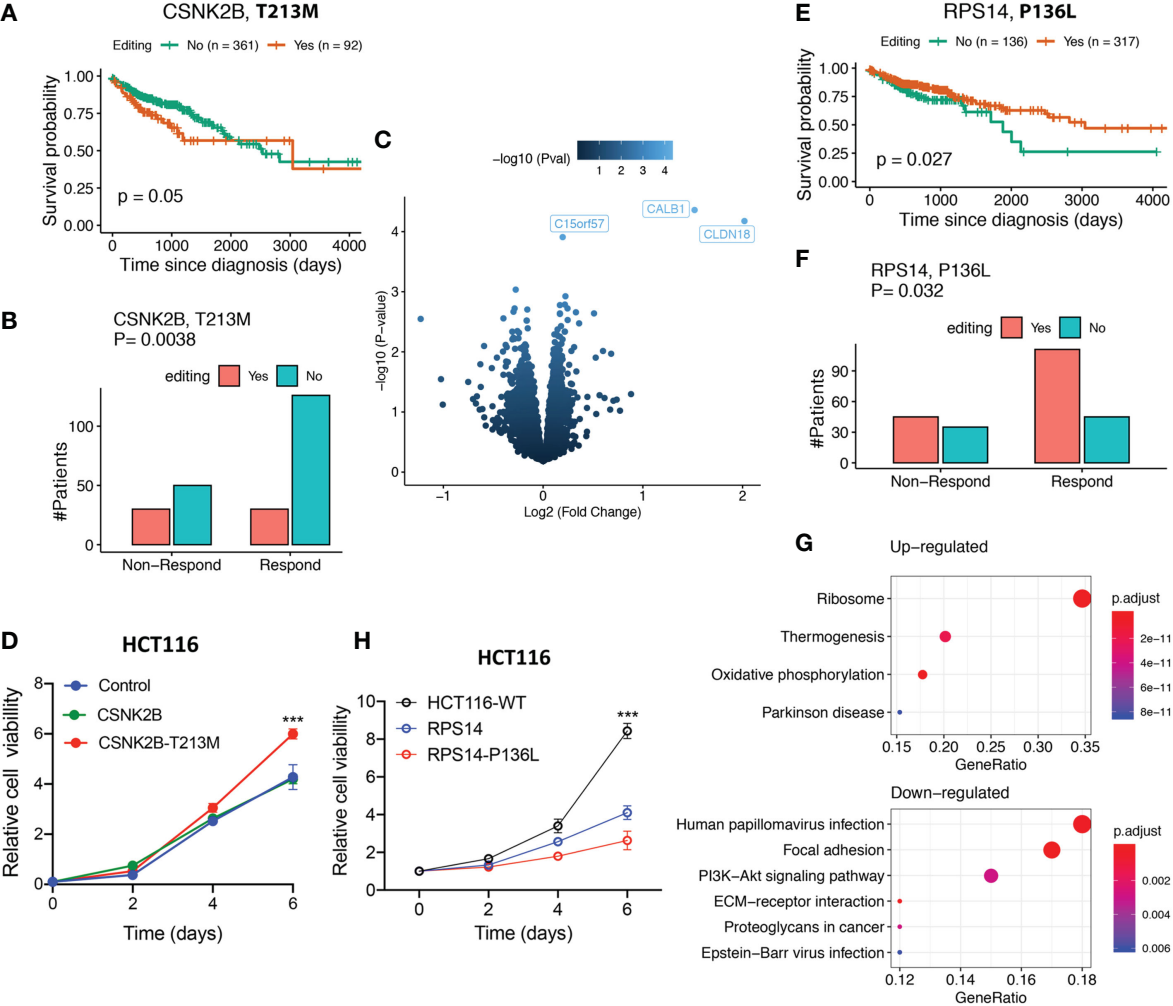
Characterization and validation of two C-to-U RESs in colon cancer. **(A)** Patients with *CSNK2B* editing showed worse survival (log-rank test). **(B)** Patients with *CSNK2B* editing showed a worse response to drug treatments (chi-squared test). **(C)** Oncogenic *CLDN18* expression level were upregulated in patients with *CSNK2B* editing. **(D)** Experimental validation of *CSNK2B* editing that promoted cell proliferation in colon cancer. **(E)** Patients with *RPS14* editing show a better survival (log-rank test). **(F)** Patients with *RPS14* editing show better response to drug treatments (chi-squared test). **(G)** Ribosome pathway upregulation was highly enriched in samples with *RPS14* editing (top). In contrast, several oncogenic pathways, including PI3K-Akt signaling, were downregulated (bottom). **(H)** Experimental validation of *RPS14* editing that suppressed cell proliferation in colon cancer. **(D, H)** Error bars indicate standard errors and P-values were calculated using the Student t-test (n=3, ***p < 0.001).

The first C-to-U site (chr6:31669916) resulted in a T213M mutation in *CSNK2B* (threonine mutated to methionine, near the C-terminal), which encodes a 215-amino acid (aa) protein as the regulatory subunit of casein kinase II (CK2), a tetrameric serine/threonine protein kinase that is upregulated in various cancers (34). We found that *CSNK2B* editing preferred late stages (*P*-value= 0.028, odds-ratio= 1.75, Chi-squared test) and was associated with worse survival of patients (**Figure 5A**) and lower drug sensitivity (**Figure 5B**) (see **Methods**). Moreover, we used SDM2 (35) to predict the editing effect on *CSNK2B* and found that the CK2 holoenzyme stability was increased by 0.3 unit energy. Because CK2 has been shown to directly regulate Wnt signaling (34), increased CK2 stability *via* CSNK2B editing may increase Wnt signaling activity. Moreover, *CLDN18* (claudin 18), encoding a tight cell junction component, was the most significantly upregulated gene in patients with *CSNK2B* editing (*P*-value=6.6e-5, Fold-change=4.0; **Figure 5C**). *CLDN18* is overexpressed in some malignant cancers, like colon cancer and gastric cancer. It is a promising biomarker for targeted cancer immunotherapy, like chimeric antigen receptor T cells (CAR-T) (36).

We introduced wild-type CSNK2B cDNA and mutant CSNK2B_T213M cDNA into HCT116 cells in order to prove that editing CSNK2B can cause cancer. We discovered that altered CSNK2B T213M considerably increased cell viability relative to wild-type CSNK2B (**Figure 5D**), implying that edited CSNK2B can contribute significantly to tumor proliferation.

The other C-to-U editing (chr5:150444335) resulted in a P136L mutation of *RPS14* (proline to leucine, also near the C-terminal). In contrast to *CSNK2B* editing, it was associated with better patient survival (**Figure 5E**) and higher drug sensitivity (**Figure 5F**). This editing increased the 40S ribosome subunit stability by 2.37 unit energy (large) after editing, as predicted by SDM2 (35). Enhanced stability of the ribosome may help better resist cellular stresses and prevent ribosomopathies, in which damaged ribosome subunits dysregulate translational homeostasis, and this eventually induces diseases including cancer (37). We found it interesting that the ribosome pathway was significantly upregulated (adjusted P-value = 2.4E-35, **Figure 5G**) in RPS14-edited samples. This may have increased the production of ribosomes and decreased cellular stress while downregulating oncogenic pathways like PI3K-Akt.

Similarly, we validated the tumor-suppressive *RPS14* editing in HCT116 colon cells. We found that edited RPS14_P136L significantly decreased cell viability compared to wild-type RPS14 (**Figure 5H**). This means that editing RPS14 stopped the growth of tumors.

## Discussion

Thousands of RNA editing sites in human coding regions have the potential to alter protein functions. In cancer, A-to-I RESs, but not C-to-U, have been demonstrated to contribute to cancer stemness (6) and protein diversity (4), and determine immunotherapy response (8). In this study, we comprehensively characterized C-to-U RESs across >20 cancer types. To our best knowledge, this is the first systematic study investigating C-to-U RESs across cancers.

One important feature of pan-cancer C-to-U RESs was that the same subset of RESs were associated with multiple features, including patient survival, cancer stemness, TMB, and ICCs, suggesting RES-mediated close relationships among these features. For example, GALM (or IFI6) editing sites associated with worse outcomes of LGG patients resulted in higher expression of GALM (or IFI6), and were correlated with higher cancer stemness. Moreover, ICC-associated RESs in *CAVIN1* or *VWA8* were also associated with lower TMB in PRAD or higher TMB in THYM, respectively. Interestingly, a large proportion of these significant RESs were identified in LGG, despite moderate nRESs in LGG. This observation further supports the critical role of C-to-U editing in the brain (3).

The outstanding associations between C-to-U RESs across different cancer types and different ICCs were also impressive, for example, *CAVIN1* RESs with CD4+ T cells or M2 macrophages, and *VWA8* with M1 or M2 macrophages. This prompted us to further look into a possible connection between C-to-U RESs and anti-PD1 immunotherapy. As a result, we identified one anti-PD1-associated RES in *CST3*, which was confirmed as a critical modulator of anti-PD1 treatment, and two associated RESs in *TPI1* and *TNC1*, with certain evidence supporting their important roles in immune checkpoint blockade treatment. We believe that further examining the RESs less significant or supported by only one anti-PD1 dataset would provide more novel biomarkers of anti-PD1 treatment. This can also be used to survey RESs related to other immune checkpoint blockade therapies, such as anti-CTLA4.

In conclusion, our analysis provides a systematic assessment of C-to-U RESs across cancer types and illuminates their functional roles, underpinning many cancer characteristics. This work would promote further investigation into C-to-U RESs by the RNA editing research community.

## Materials and methods

### RES identification and annotations for RNA-seq datasets

For all RNA-seq samples, except for anti-PD1 samples, C-to-U RESs were detected by applying the single nucleotide polymorphism (SNP)-independent algorithm SPRINT (18) with default parameters to BAM files based on hg38. For anti-PD1 RNA-seq samples, C-to-U RESs were detected by applying REDItools (38) to hg38 BAM files created by STAR (39). To improve the quality of RESs, they were filtered as follows: (1) >=2 reads supported editing; (2) >=10 reads in total covered each RES; (3) sites with 100% editing level removed; (4) >=3 (or >=1%) samples detected editing in each cancer; (5) not overlapping SNPs with variant allele frequency>=0.1 for anti-PD1 RESs. RESs were annotated by ANNOVAR (40) with gene symbols, genomic regions, and dbSNP150 allele frequencies.

### Differential editing analysis for tumor features

The following tumor information or features were downloaded from the Xena (41) database: clinical data including survival information, cancer stemness score, TMB, and immune subtypes. Tumor-infiltrated ICCs were obtained from GEPIA2021 (42). Anti-PD1 treatment response information was obtained from corresponding publications (43, 44). For each RES, samples were separated into two groups based on editing existence for comparison of survival, stemness, TMB, and ICCs. Pearson correlation between nRES and stemness scores was conducted in each cancer. Pearson correlation between ICCs with nRES or average editing levels was also conducted in each cancer. The average editing level difference across immune subtypes was examined by the Kruskal-Wallis test. Chi-squared test was performed to examine the anti-PD1 treatment difference between responsive and non-responsive (or progressive disease) patients. TCGAbiolinks (45) were used for patient survival difference analysis. We also used Cox multivariate regression to manually examine the effect of confounding variables including age, gender, stage, tumor purity, and ploidy.

### Selection of novel functional RESs in colon cancer

Only missense or nonsense RESs that are more likely to be functional were kept. Although RESs called by SPRINT were not confounded by SNPs (18) and some reported SNP sites were actually RESs (46), we still filtered out possible SNPs from RESs. Next, RESs overlapping reported sites in RADAR (1) database were removed. Only RESs with support from at least four samples were kept. Furthermore, RESs were required to be supported by mass spectrometry-based proteomic peptides from the Clinical Proteomic Tumor Analysis Consortium (CPTAC) (33) (see MS peptide searching). Similar to a previous study (4), we re-examined the raw sequencing reads for all samples by counting high-quality reference and edited bases to obtain the editing status in each sample for the remaining RESs. Then, using TCGAbiolinks (45), we compared the survival difference of patients with or without editing for each RES and kept only RESs showing nominal significance (P <= 0.05) of differential survival. Lastly, RESs that overlapped with possible somatic mutations in patients were manually examined and excluded.

### MS peptide searching

Wildtype and edited protein sequences for RESs were combined with reference protein sequences from UniProt and translated protein sequences from RefSeq, GENCODE, and Ensembl to comprise the search database for MS proteomics data. MS_Pycloud (https://bitbucket.org/mschnau/ms-pycloud) was used to search against this database for peptide matches with a false discovery rate (FDR) < 0.01. Resulting peptides were further processed to identify novel peptides that appeared only after specific editing events.

### Differential gene expression analysis

Differential gene expression analysis in terms of editing status was performed by the limma R package (47), variables including age, sex, race, stage, subtype, tumor purity, and ploidy were adjusted. KEGG pathway enrichment of differential results (adjusted P-value < 0.1) were analyzed and visualized using clusterProfiler (48). Processed gene quantification results for TCGA-COAD were obtained from (49).

### Drug response analysis

Patient treatment responses were classified into responders (Complete Response or Partial Response) and non-responders (Progressive Disease). For each RES, patients were grouped by editing status. Then, a chi-squared test was used to compare the difference of the responses between patients with or without editing.

### Protein stability prediction

Computational prediction of editing impact on protein stability was performed by Site Directed Mutator (SDM2) (35) using experimentally resolved 3D protein structures from Protein Data Bank (PDB). The 3D structure accession number is 4DGL for CSNK2B and 6G53 for RPS14.

### Mammalian cell culture and stable cell lines

HCT116 and HEK293T cells were cultured at 37°C/5% CO2 in DMEM (Hyclone) with 10% fetal bovine serum (Hyclone) and 1% penicillin-streptomycin (GIBCO). For the generation of HCT116 stable expression cells, the constructs were transfected into 293T cells for retroviral packaging and subsequent transduction. Stable expression cells were selected by puromycin selection.

### Cell viability assay

The HCT116 CSNK2B or RPS14 (wild type or editing mutation) stable cell lines were seeded into 96-well plates, and the assays were performed at day 2, 4, and 6 time points. Cell viability was detected with CellTiter-Glo 2.0 (Promega) according to the manufacturer’s instructions. The significance of the differences was analyzed with the Student’s t-test.

## Data availability statement

The original contributions presented in the study are included in the article/**Supplementary Material**. Further inquiries can be directed to the corresponding author. RNA-seq data, somatic mutations, and clinical information for 375 TCGA samples were downloaded from the TCGA Data Portal (https://portal.gdc.cancer.gov). CPTAC data was downloaded from https://proteomics.cancer.gov/programs/cptac.

## Author contributions

MG and YX conceived the project and designed the study. MG performed the analyses with the assistance of ZF. MG interpreted the results. FL performed experimental validation. MG wrote the manuscript. LZ, HY, and ZS contributed scientific input. YX and HY reviewed the manuscript. All authors contributed to the article and approved the submitted version.
