# Supplementary material for: Pan-cancer investigation of C-to-U editing reveals its important role in cancer development and new targets for cancer treatment
Source: Front Oncol. 2023 Mar 9;13:1097667. doi: 10.3389/fonc.2023.1097667 (PMC10034049; doi:10.3389/fonc.2023.1097667)
Supplement: Supplementary file 2 [file DataSheet_1.docx]

**Supplementary Figures and Legends**


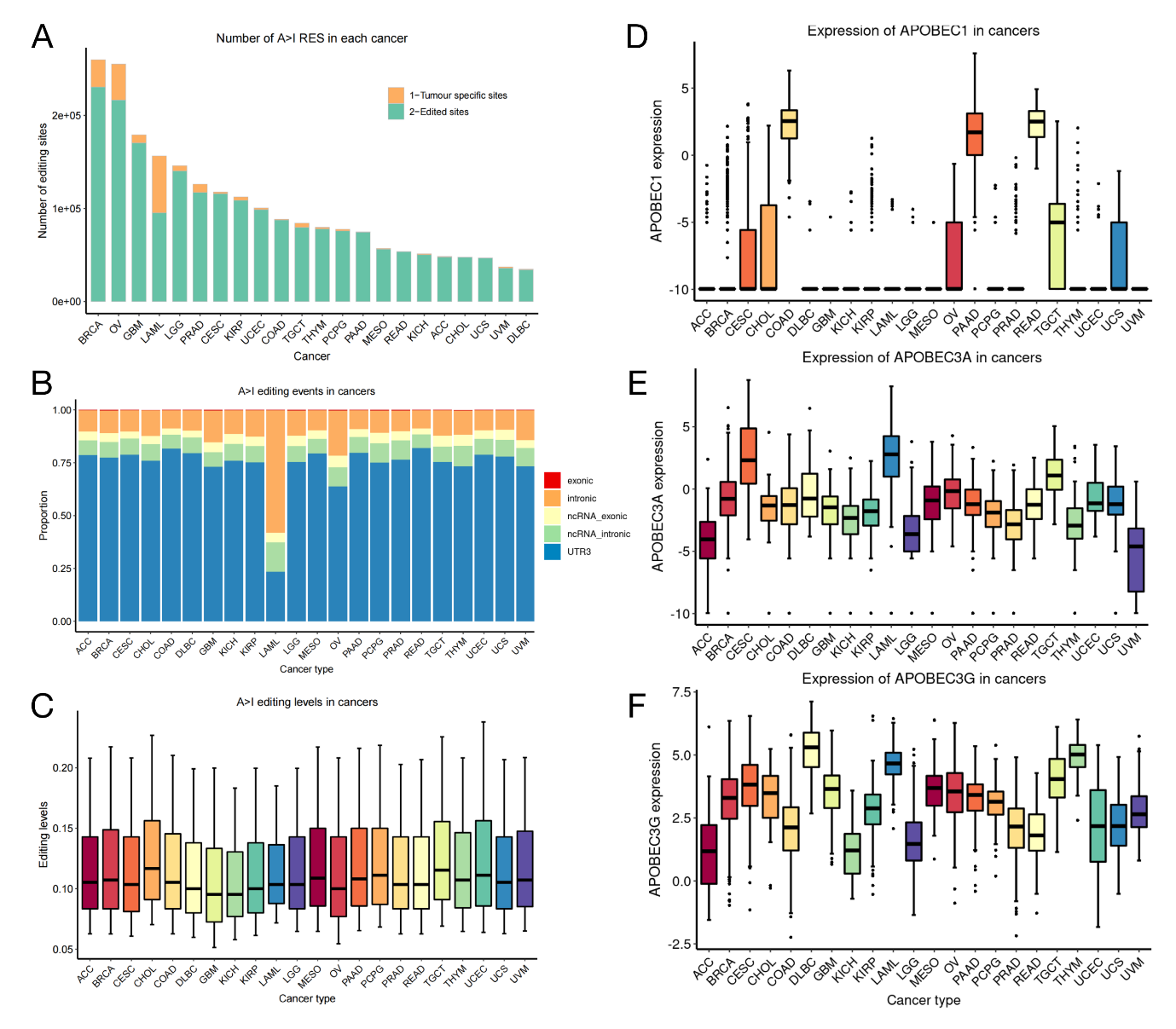


**Fig. S1, Overview of pan-cancer A-to-I RESs and expression of APOBEC enzymes.** (**A**) Number of RESs across cancer types. The orange color indicates tumor-specificity. (**B**) Genomic region distribution of RESs across cancers. LAML was derived from blood, in contrast to other solid tumors. (**C**) Average editing levels of RESs across cancers. (**D**) Expression levels of *APOBEC1*, *APOBEC3A*, and *APOBEC3G* across cancers.

**Fig. S2**, Correlations between EL and nRES in 22 tumors.

**Fig. S3**, Different editing levels across immune subtypes in BRCA, PCPG, TGCT, and UCS.

**Supplementary Tables**

**Table S1, C-to-U RESs associated with patient survival of various cancers.**

**Table S2, C-to-U RESs associated with ICCs of various cancers.** RESs also associated with TMB were indicated in the last column.
